# Philanthropic donor perspectives about providing harm reduction services for people living with HIV/AIDS in a hospital setting

**DOI:** 10.1186/s12954-022-00711-8

**Published:** 2022-11-16

**Authors:** Katherine Rudzinski, Soo Chan Carusone, Andre Ceranto, Francisco Ibáñez-Carrasco, Lisa McDonald, Dean Valentine, Adrian Guta, Elaine Hyshka, William O’Leary, Andra Cardow, Carol Strike

**Affiliations:** 1 School of Social Work, University of Windsor, 167 Ferry Street, Windsor, ON N9A 0C5 Canada; 2 Dalla Lana School of Public Health, University of Toronto, 155 College St., Toronto, ON M5T 3M7 Canada; 3 Casey House, 119 Isabella St, Toronto, ON M4Y 1P2 Canada; 4 McMaster Collaborative for Health and Aging, McMaster University, 1280 Main Street West, Hamilton, ON L8S 4K1 Canada; 5 School of Public Health, University of Alberta, 3-256 Edmonton Clinic Health Academy, 11405 - 87 Ave NW, Edmonton, AB T6G 1C9 Canada; 6 Lyle S. Hallman Faculty of Social Work, Wilfrid Laurier University, 120 Duke St W, Kitchener, ON N2H 6P6 Canada

**Keywords:** Philanthropy, donors, harm reduction, supervised consumption services, hospital-based harm reduction services, mixed methods research, people who use drugs, people living with HIV/AIDS

## Abstract

**Background:**

Hospital-based harm reduction services are needed to reduce drug-related harms, facilitate retention in care, and increase medical treatment adherence for people who use drugs. Philanthropic donor support plays a key role in delivering such innovative services which might fall outside current funding streams. However, little is known about how the principles, implementation, and practice of harm reduction services, which are often highly stigmatized, may impact donor behaviours. We explored this issue within Casey House, a speciality hospital in Toronto, Canada.

**Methods:**

Our mixed methods study utilized an explanatory sequential design. A convenience sample of *n* = 106 philanthropic individual donors, recruited via email, completed an anonymous web-based survey, between July and October 2020, which assessed their knowledge of harm reduction services and the potential impact of implementing new hospital-based harm reduction services on donors’ future support. Following this, we conducted semi-structured qualitative interviews with *n* = 12 of the donors who completed a survey and volunteered to be interviewed. Interviews examined donors’ perspectives about harm reduction and their hopes/concerns for such programming at Casey House. Data were analysed using descriptive statistics and participatory-based thematic analysis.

**Results:**

Survey data show a high level of support for hospital-based harm reduction services, with participants reporting that they “strongly agree/agree” with providing harm reduction equipment (85%), supervised consumption services (82%), and prescription opioid treatment (76%) at Casey House. A majority of participants (66%) claimed that implementing new harm reduction services at the hospital would not impact their future donation, while 6% said they would be less inclined to donate. Interview participants were supportive of harm reduction services at Casey House, recognizing the benefits of providing such services for hospital clients and the wider community. However, some spoke of the potential impact that implementing hospital-based harm reduction services may have on “other” donors who might be opposed. Although some believed harm reduction services should be fully funded by the government, most saw a role for donors in supporting such services.

**Conclusions:**

Our findings show support of hospital-based harm reduction services among philanthropic donors and provide insight into how donor support may be affected when such services are introduced.

**Supplementary Information:**

The online version contains supplementary material available at 10.1186/s12954-022-00711-8.

## Background

Hospital foundations play a key role in securing financial assistance, through fundraising initiatives and donor engagement, for hospitals [1, 2]. Donor dollars, which come from both individual donors and organizations, are typically used by hospitals to enhance patient care, assist with capital projects, and fund new services [1, 3]. Philanthropy is quickly becoming a crucial source of support for health care organizations. A 2018 survey of 123 academic medical centres in the USA showed that health care organizations raised nearly $2.7 billion (an average of $21.8 million per hospital) from non-staff and non-alumni individual donors, primarily consisting of former/current patients and their families, in just one year [4]. Donor support plays an important role in delivering novel services which might fall outside regular funding streams. Harm reduction programs (e.g. supervised consumption services (SCS), naloxone training), which are aimed at reducing drug-related deaths and harms [5], often require such patronage due to the slow and inconsistent government support of these services [6, 7].

The ongoing North American opioid overdose crisis, resulting in 30,843 deaths in Canada between January 2016 and March 2022 [8] and over 340,000 deaths in the USA in that time frame [9, 10], and the current coronavirus (COVID-19) pandemic have created an urgent need for harm reduction services [11, 12]. However, even though these services preserve public health and safety, are cost-effective, and provide life-saving supports for people who use drugs (PWUD), they remain underfunded and under-implemented [6, 13], especially in hospital settings. Conservative estimates show that between 30 and 45% of PWUD continue to use drugs while in hospitals, where standard abstinence-based policies often lead to compulsory discharges and high rates of patients leaving against medical advice, which can result in disruptions to medical treatment, increased morbidity and mortality, and increased drug-related harms [14–18]. Hospital-based harm reduction services, including SCS, have been increasingly recommended, and in a few cases implemented, as a valuable complement to existing healthcare services for PWUD [19–23]. However, little is known about how hospital-based harm reduction services may impact broader organizational operations, like fundraising or donor support.

Although much is known about charitable giving [24–26], research about donation specifically for harm reduction services is lacking [27]. Charitable behaviour is multifaceted and driven by a variety of factors. Typically donors select causes that resonate with their values, personal experiences, and desire to make a positive impact [26, 28, 29]. Recently, the challenges of fundraising for socially stigmatized populations (e.g. people with substance use disorders, ex-offenders, sex workers), often referred to in the philanthropy literature as “unpopular” causes, have been gaining attention [28, 30]. Some studies show that fundraising for the well-being of “sympathetic” or “deserving” groups is easier than engaging donor support for groups often seen as responsible for their problems or those who are often blamed for social ills [30, 31]. Although there is evidence of successful fundraising campaigns for issues such as mental health, HIV (human immunodeficiency virus), AIDS (acquired immunodeficiency syndrome), and addiction treatment (e.g. MAC AIDS Fund, Elton John AIDS Foundation, The National Alliance on Mental Illness’ NAMI Walks Your Way Campaign, the Center for Addiction and Mental Health’s Today Campaign) [28, 32–35], fundraising for harm reduction services has not been well researched. Given the ongoing political, legal, and moral opposition to harm reduction, and the deeply ingrained stigma against PWUD [6, 13], this may inhibit donations for hospital-based harm reduction programs. Indeed, hospital fundraisers may be concerned that asking for support for harm reduction services or even acknowledging that some clients use drugs may jeopardize existing donations. However, little is known about how current donors perceive this or how the principles, implementation, and practice of in-hospital harm reduction services may impact their future support.

We set out to examine donor perspectives about hospital-based harm reduction services for people living with HIV/AIDS at Casey House, a speciality HIV hospital in Toronto, Canada. The need for comprehensive and coordinated care is particularly important for people living with HIV/AIDS who use drugs [36, 37]. Implementation of in-hospital harm reduction services can prevent overdose deaths, as well as facilitate retention in care and increase adherence to antiretroviral medication, thereby improving the HIV treatment cascade [38, 39]. The goal of this mixed methods study was to explore Casey House donor opinions about various types of harm reduction programming and investigate how, if at all, the implementation of further hospital-based harm reduction services may impact donor’s future support.

## Methods

Casey House is a sub-acute care specialty HIV hospital in Toronto serving people living with, or at-risk of, HIV who are frequently also dealing with medical complexity and social vulnerability (e.g. mental health issues, substance use disorder, cognitive impairment, poverty, and unstable housing) [40]. The hospital includes a 14-bed inpatient unit, which sees 100 + yearly admissions providing sub-acute care, respite, and palliative care, and an outpatient day health program assisting approximately 350 clients with a combination of one-on-one and group programs (e.g. lunch program, clinical groups, social/recreational programming). Clients frequently move between outpatient and inpatient units in response their health and care needs.

Since 2014, Casey House has been the primary distributor of harm reduction kits to clients and PWUD in this neighbourhood [41]. An estimated 80% of Casey House clients use substances, and the surrounding neighbourhood has the most emergency medical service overdose calls in the city [42]. Building on support from a recent feasibility study [19], in 2021 Casey House received a federal exemption under section 56.1 of the Controlled Drugs and Substances Act [43] to provide SCS for clients. The hospital did not apply for additional government funds to operate the services: capital expenses and additional staffing would be supported with a grant from the hospital’s foundation. With this new need for annual funds for harm reduction services, and as the hospital continues to implement more such programming, there is a desire to know how, if at all, these changes will impact donor support.

This mixed methods study included an online survey and follow-up qualitative telephone interviews with Casey House donors. The survey was created using Qualtrics web-based software, and included questions about socio-demographic (e.g. age, gender, ethnicity) information and donation activities/experiences supporting Casey House (e.g. length of time as donor, level of donation). The survey also assessed participant knowledge of, and opinions about, harm reduction and the potential impact implementing new hospital-based harm reduction services would have on donors’ future support.

In July 2020, an invitation with a link to the short anonymous web-based quantitative survey was emailed to 1434 recent hospital donors (individuals who had donated at least once in past 36 months) and 1285 received the email. The survey was open until mid-September 2020 and all donors received at least one reminder email. A total of 127 donors responded to the survey link: *n* = 8 did not consent to participate; *n* = 13 started but did not complete the survey; *n* = 106 completed the survey. Of those who received the email invitation, 8.2% completed the survey.

A total of 20 survey participants provided contact information for a qualitative interview. We selected 12 donors to interview that had a breadth of opinions (supportive, undecided) about harm reduction and different giving experiences (length of time as donor; level of donation). Qualitative interviews were conducted by phone with donors in October and November 2020 and lasted approximately 30–60 min.

We asked survey participants if they agreed or disagreed with making three different harm reduction services (i.e. harm reduction kit distribution, supervised consumption services, prescription opioid treatment) available as (a) part of overall health care in Ontario and (b) at Casey House. Donors were asked to answer on a scale ranging from strongly agree to strongly disagree. A definition of harm reduction was provided after participants had a chance to rate their self-assessed level of harm reduction knowledge and then a description of each of the three services was provided before asking donors to give their opinions (see Additional file 1 for donor survey questions and full definitions).

At the end of the survey, each participant was invited to take part in a one-on-one telephone-based qualitative interview about harm reduction services. Prior to the start of these interviews, verbal informed consent was obtained. Interview questions focused on participants’ experiences as a donor; their hopes and concerns about Casey House providing additional harm reduction services; how the addition of these services at Casey House may impact their future support; how they felt about their donation potentially supporting in-hospital harm reduction services; and, what impact they thought these programs may have on clients and the broader community. No compensation was provided for participation in the survey or the interview.

Our study used an explanatory sequential design which started with quantitative data collection (survey), followed by qualitative data collection (interviews) [44]. Survey data were analysed using SPSS 26 and focused on descriptive statistics. All missing values and non-responses, including prefer not to answer options, were removed and excluded from percentages in the descriptive statistics. All interviews were audio-recorded and transcribed verbatim. Qualitative data was coded and analysed using NVivo 12 analysis software. We compared the quantitative and qualitative findings after separate analyses and used the qualitative data to provide nuance to key quantitative findings [45]. While the survey provided us with a general understanding of the knowledge of and acceptability for harm reduction services, the qualitative data allowed us to refine and explain our statistical results by exploring donors’ views on harm reduction in more depth [46].

We used an adaptation of DEPICT, a participatory approach, wherein as a group of stakeholders with diverse perspectives (e.g. people living with HIV, staff, donors, fundraisers, researchers) we engaged in collaborative data analysis [47, 48]. Through dynamic reading, summarizing and highlighting key themes (inductive) of select transcripts, we collaboratively designed and piloted a codebook which was refined based on feedback from the wider team. We inclusively reviewed and summarized categories, selected key quotes, and engaged in concept mapping to explore themes [47, 49]. Data saturation was reached on all key categories of interest [50, 51]. This study and related protocols were approved by the University of Toronto HIV Research Ethics Board.

## Results

Most survey participant reported being 55 years old or older (71%; *n* = 74) and identified as white/ (89%; *n* = 94). Just over half identified as cis male (53%; *n* = 55) and 89% (*n* = 92) stated that they currently resided in the greater Toronto area. Of the 12 donors interviewed 75% (*n* = 9) were aged 55 or older, 67% (*n* = 8) identified as cis male, and all resided in the greater Toronto area (see Table 1). Please note that qualitative numerical results in the tables constitute a small and non-random subset of the survey respondents and as such are not equivalent to the survey data results.

**Table 1:** Socio-demographic characteristics

Participant characteristics	Survey % (*n*)	Qualitative interview % (*n*)
*Gender*		
Male	53 (55)	67 (8)
Female	46 (48)	33 (4)
Non-binary	1 (1)	0
Ethnicity*		
White	89 (94)	83 (10)
Asian	6 (6)	17 (2)
Other	5 (1)	0
*Age*		
18–34	9 (9)	8 (1)
35–54	20 (21)	17 (2)
55–74	57 (59)	67 (8)
75+	14 (15)	8 (1)
*Education (highest completed level)*		
High school or less	5 (5)	8 (1)
College or university	51 (53)	58 (7)
Graduate or professional	44 (45)	33 (4)
*Marital status*		
Married/living with partner	52 (51)	33 (4)
Single/never married	28 (28)	58 (7)
Divorced/separated	10 (10)	0
Widowed	10 (10)	8 (1)
*Residence*		
Greater Toronto Area	89 (92)	100 (12)
Elsewhere in Canada	8 (8)	0
Outside of Canada	3 (3)	0

*Could select multiple response

Survey results are clarified and expanded upon using quotes from donor interviews focusing on three main themes: donor relationship with Casey House and giving experience; knowledge of and opinions about harm reduction; and impact of in-hospital harm reduction services on future donations. An additional theme regarding the impact of the interview process will also be discussed.

### Relationship with Casey House and giving experience

Survey participants primarily identified as online donors (56%; *n* = 59), monthly donors (22%; *n* = 23), and/or donated by mail (19%; *n* = 20). Survey participants reported a range for the length of time they had donated to the hospital, with 14% (*n* = 15) identifying as first-time donor, and 15% (*n* = 16) donating for more than 20 years. For level of donation in the past 12 months, more than half of survey participants donated less than $250, while for interview participants more identified as having donated $1000+ (see Table 2).

**Table 2:** Donation characteristics

	Survey % (*n*)	Qualitative interview % (*n*)
Type of donor*		
Online donor	56 (59)	50 (6)
Monthly donor	22 (23)	25 (3)
Mail-in donor	19 (20)	8 (1)
Casey House volunteer/peer	11 (12)	17 (2)
Signature event attendee	11 (12)	33 (4)
Leadership donor	7 (7)	33 (4)
Capital campaign donor	7 (7)	25 (3)
$25,000 + donor	6 (6)	17 (2)
Telemarketing donor	3 (3)	0
*Length of time as donor*		
First-time donor	14 (15)	8 (1)
1–5 years	27 (29)	33 (4)
6–20 years	36 (38)	25 (3)
20 + years	15(16)	33(4)
Former donor	8 (8)	0
*Level of donation (past 12 months)*		
Less than $250	53 (55)	33 (4)
Between $250 -$1000	18 (19)	25 (3)
$1000 +	17 (17)	42 (5)
Did not donate in past 12 months	12 (12)	0
Reason for donating to Casey House*		
Believe in hospital and its cause	89 (94)	92 (11)
Feel compassion for PLHIV and desire to contribute to community	73 (77)	83 (10)
Casey House has helped or is helping family member or friend	18 (19)	33 (4)
Live close to Casey House	17 (18)	25 (3)
Tax benefit	13 (14)	33 (4)
Volunteer/peer at Casey House	11 (12)	17 (2)
Another reason (e.g. in memoriam, personal connection to hospital)	19 (20)	8 (1)
*How well they felt their support was recognized and appreciated by Casey House*		
Extremely	67 (68)	58 (7)
Somewhat	27 (27)	42 (5)
Neutral	4 (4)	0
Not very	2 (2)	0

*Could select multiple responses

The top two reasons for donating to Casey House reported by survey participants were because they believed in the hospital and its cause (89%; *n* = 94) and/or they felt compassion for people living with HIV/AIDS and had a desire to contribute to this community (73%; *n* = 77). Other reasons for donating included Casey House helping a family member/friend (18%; *n* = 19), living close to the hospital (17%; *n* = 18), tax benefits (13%; *n* = 14), and identifying as a Casey House volunteer or peer (11%; *n* = 12) (see Table 2).

Many interview participants spoke about a personal connection to Casey House—in that a family member, significant other, or friend had received care at the hospital, and for some this evolved into recognizing the “crucial need” for services that the hospital provides:

*I think I originally started because I wanted to support them as a result of the great care that they have taken of my friend. But I think it sort of morphed from there, and the more I read about them and the work that they did, I think they fill an important need in downtown Toronto.* [DS115]

Others felt a connection to Casey House because they identified as lesbian, gay, bisexual, transgender, or queer (LGBTQ+) and wanted to support this community with their donation, as one participant explained:

*It's important for me to sort of give back to the LGBT community, in particular as a gay man, the aspect around HIV at that point in time was something that sort of affected the community very, very hard. So I felt I needed to sort of give back… Probably about ten years after I started helping out… I actually seroconverted myself. So, it's still a matter of being able to give back to the community, but there's a little bit more of a personal aspect to it now, as opposed to before it was – I'm there, because it's part of my community. Now it's not only just a part of my community, it's a part of me as well.* [DS114]

For some, donating to Casey House mattered because the organization was providing essential services for a community that can be marginalized in other healthcare settings. Donors felt this was meaningful because they were joining forces to support social change they believed in. As one participant explained, *I just feel like that, they're* [Casey House] *fulfilling a need of people who fall through the cracks in the public healthcare system* [DS078]. Another participant stated:

*A lot of people that use Casey House services kind of have a marginalized experience in the community… That non-judgmental attitude and the support that's provided* [by Casey House]*, despite any of those things, is wonderful… It does really good work, with the community they serve. I know it's a model, internationally, for providing this type of support. It's wonderful to have that in our city, and to be, you know, in my small way, being a part of it. I'm quite pleased.* [DS094]

### Knowledge of and opinions about harm reduction

Self-reported knowledge of harm reduction varied with 27% (*n* = 29) of survey participants stating that they had very little to no knowledge, 24% (*n* = 25) reporting some knowledge, and 23% (*n* = 24) average knowledge. Some survey participants claimed to be fairly knowledgeable (16%; *n* = 17) and some very knowledgeable (10%; *n* = 11). No interview participants reported very little or no knowledge of harm reduction, half identified as fairly or very knowledgeable (see Table 3), and some shared stories about family or friends who had struggled with substance use disorders. A few interviewees cited lived experience of substance use disorders, which helped to shape their opinions. As one participant stated*, I have been in recovery from drug and alcohol issues for about thirteen, fourteen years now. I have personal experience with drug abuse. And I am sensitive to and aware of…the need for a robust suite of services available to people who are struggling.* He went on to explain, *so why do I support the harm reduction services in Casey House? Because I have personal knowledge of how useful, helpful and life-saving harm reduction services can be in the community* [DS056].

**Table 3:** Self-reported level of knowledge about harm reduction

Level of harm reduction knowledge	Survey % (*n*)	Qualitative interview % (*n*)
No/very little knowledge: *I have not heard the term before or I know the term, but not a lot about what is included*	27 (29)	0
Some knowledge: *I know something about the services or programming offered*	24 (25)	25 (3)
Average knowledge: *I know something about programming/ services offered and about evidence of the effectiveness of harm reduction*	23 (24)	25 (3)
Fairly knowledgeable: *I know details about programming/services offered and evidence about the effectiveness of harm reduction, have followed the news about setting up new harm reduction services in Toronto/across Canada*	16 (17)	33 (4)
Very knowledgeable: *I know details about programming offered and evidence supporting harm reduction, have followed the news about setting up new harm reduction services, have done some extra reading or know people who work at or have used a harm reduction program/service*	10 (11)	17 (2)

Survey data showed a high level of support for hospital-based harm reduction services, as well as for these services being provided as part of overall healthcare in Ontario. Support for provincial services was slightly higher for harm reduction kit distribution and supervised consumption services, while prescription opioid treatment got more support as an in-hospital service at Casey House (see Table 4). Survey participants reported that they strongly agree/agree with providing harm reduction equipment (85%; *n* = 88), SCS (82%; *n* = 85), and prescription opioid treatment (76%; *n* = 79) at Casey House.

**Table 4:** Donor opinions on harm reduction services in Ontario and at Casey House

Harm reduction service	Opinion	Agree/disagree with providing service as part of overall health care in Ontario		Agree/disagree with providing service at Casey House	
		Survey % (*n*)	Qualitative interview % (*n*)	Survey % (*n*)	Qualitative interview % (*n*)
Harm reduction kit distribution	Strongly agree/agree	88 (91)	92 (11)	85 (88)	83 (10)
	Undecided	11 (11)	8 (1)	10 (10)	17 (2)
	Disagree/strongly disagree	1 (1)	0	5 (5)	0
Supervised consumption services	Strongly agree/agree	86 (89)	92 (11)	82 (85)	92 (11)
	Undecided	10 (10)	8 (1)	11 (11)	8 (1)
	Disagree/strongly disagree	4 (4)	0	7 (7)	0
Prescription opioid treatment	Strongly agree/agree	73 (76)	83 (10)	76 (79)	92 (11)
	Undecided	22 (23)	17 (2)	18 (19)	8 (1)
	Disagree/strongly disagree	5 (5)	0	6 (6)	0

Some interview participants perceived harm reduction services as a natural extension of the care that Casey House is already providing and in line with the hospital’s perceived mission as *a compassionate centre for care of people who are marginalized* [DS053]. As one donor explained: *I think this is part of an essential service, from my perspective… and a natural outgrowth of what they've been doing… I think most people would understand that* [DS115].

While others believed that Casey House was the ideal place for offering harm reduction services, due to the structure of programming and other services already provided at the hospital:

*I'm going to qualify this by saying that I'm not a healthcare worker. I don't know a ton about harm reduction. But what I do know, suggests to me that it works best within a system of care. And, given, again, that Casey House supports this client base that has so many overlapping issues, I think it makes perfect sense for harm reduction services to be integrated as part of the wider care that they provide.* [DS069]

Most donors perceived benefits of providing harm reduction services for Casey House clients, and the wider community, including increased safety, well-being, and decreased healthcare costs:

*It's part of the puzzle, and I think to ignore it is foolish… It's not ideal that people are addicted to drugs, but I think it's important to realize that's the case… So anything that they can do to reduce the risk of overdose or violence or anything like that, I'm all for it.* [DS053]

*If you have people who are drug users who [are] in better health… who eat better because the harm reduction has made it possible to do things, to take better care of themselves, who are less exposed to infections that are transmitted by sharing needles, who are more able… to follow a recommended course of healthcare… then presumably all of that would mean that you'd be spending less down the road on healthcare costs.* [DS094]

A number of interview participants acknowledged the value of integrating harm reduction services in hospital, especially in their capacity to retain clients in care:

*I think some people feel they have to make a choice between their use, and the care they receive. So combining those services in the same agency, I think would, as I said, it's not just about saving lives, but it's also like the quality of their lives, while they're… using… I see the real connection there myself. And I think that would probably, be a benefit to clients and patients overall.* [DS105]

Approximately 10% of donors surveyed were undecided about providing kit distribution or SCS, but those numbers rose to around 20% for prescription opioid treatment. A small number (less than 7%) of donors disagreed/strongly disagreed with providing these services in-hospital (see Table 4). Among survey participants who disagreed/strongly disagreed with harm reduction services none volunteered to do an interview. Three interview participants were undecided about providing at least one of the services asked about in the survey. In spite of this, interview participants discussed potential concerns of introducing hospital-based harm reduction services, including concerns over client and staff safety from potential “violent” or “aggressive” behaviours of clients using SCS, and potential pushback from neighbours with a not-in-my-backyard (NIMBY) attitude towards introducing harm reduction services in the community. Another participant explained concerns over potential service disruption to clients who do not use drugs:

*There is going to be …exposure… to seeing heavy drug use…. So, if you expose those kinds of people to the people that are not drug users, in a fragile state, it may become fearful for them. … And it may affect how they want to stay. Maybe they would not want to complete the program, because they feel uncomfortable in a place that is having heavy drug users come into the facility. I don't know how they're going to manage that. But I think that has to be managed.* [DS053]

Some donors also talked about their personal journeys to understanding harm reduction and developing empathy for people dealing with substance use disorder. Some mentioned growing up in religious or conservative families where addiction was viewed “as a weakness” or as a moral “choice” and they had little exposure to PWUD. Others declared that the attitudes or values they were raised with meant that they had “blinders on” when it came to their view of substance use. As one participant explained, *but for some people… it's part of who they are. Like, it's their identity, 'I'm a Roman Catholic. Drugs are wrong.' Like, that's their attitude, right?* Indeed, others agreed that value and attitudes may be difficult to change, and for many these were only impacted slowly over time and through personal experience and education. As one participant explained:

*To clarify, I grew up in a house where, you know, alcohol particularly was a problem… I had some other relatives for whom hard drugs were [...] I'm talking about booze, heroin […]. I grew up at a time when this behaviour was demonized and people were kind of labelled… 'This is who they are.' And… through my education and mostly my work experience, and the hundreds of men I've worked with over the years,* [I’ve learned] *that nothing's that simple.* [DS105]

### Impact on future donation

A majority of survey participants (66%; *n* = 69) stated that implementing new harm reduction services at Casey House would not impact their future donation; 22% (*n* = 23) said they would be more inclined to donate, while only 6% (*n* = 6) said they would be less inclined. No one who felt they would be less inclined to donate or were undecided about future donations volunteered to do an interview (see Table 5).

**Table 5:** Potential impact of hospital-based harm reduction services on donors’ future support

Impact	Survey % (*n*)	Qualitative Interview % (*n*)
No impact on donation	66 (69)	67 (8)
More inclined to donate	22 (23)	33 (4)
Less inclined to donate	6 (6)	0
Undecided	6 (6)	0

For survey participants who were more inclined to donate or undecided about future donation (*n* = 29), the top reasons why they might donate more focused on the benefits of harm reduction services, including that these services encourage safer drug use (90%; *n* = 26); reduce overdose deaths and infectious disease (79%; *n* = 23); increase contact of clients who use drugs with health and social workers (69%; *n* = 20); help to retain hospital clients in HIV care (62%; *n* = 18); and reduce crime and neighbourhood problems (45%; *n* = 13). Moreover, survey participants also said that the overdose crisis warrants harm reduction at Casey House (76%; *n* = 22); harm reduction is in line with the care Casey House should provide (65%; *n* = 19), and hospital/outpatient programs are ideal locations for harm reduction (59%; *n* = 17) (see Table 6).

**Table 6:** Reasons why participants may be undecided or more inclined to donate

Reason*	Survey % (*n* = 29)	Qualitative Interview % (*n* = 4)
Harm reduction services encourage safer drug use	90 (26)	100 (4)
Harm reduction services reduce overdose deaths and infectious disease transmission	79 (23)	100 (4)
Overdose crisis warrants harm reduction at Casey House	76 (22)	75 (3)
Harm reduction increases contact of clients who use drugs with health and social workers	69 (20)	75 (3)
Harm reduction is in line with the care I believe Casey House should provide	65 (19)	75 (3)
Harm reduction services help to retain Casey House clients in HIV care	62 (18)	50 (2)
A hospital/outpatient programs are ideal locations for harm reduction	59 (17)	50 (2)
Harm reduction reduces crime and neighbourhood problems	45 (13)	0
Other	10 (3)	25 (1)

*Could select multiple responses

For those survey participants who were less inclined to donate or undecided (*n* = 12) the reasons why they might donate less included: that harm reduction services would take away from the focus on HIV/AIDS at Casey House (75%; *n* = 9); these services were out of line with the care that Casey House should provide (68%; *n* = 7); harm reduction increases crime and other neighbourhood problems (25%; *n* = 3); and a hospital is not a place for harm reduction (17%; *n* = 2). Half of these participants (*n* = 6) thought that providing harm reduction services would disrupt care for Casey House clients who do not use drugs and one-quarter (*n* = 3) thought harm reduction services could cause liability issues for the hospital (see Table 7).

**Table 7:** Reasons why participants may be undecided or less inclined to donate

Reason*	Survey % (*n* = 12)
Harm reduction services will take away from the focus on HIV/AIDS at Casey House	75 (9)
Harm reduction services are out of line with the care and services that I believe Casey House should provide	68 (7)
Providing harm reduction services would disrupt care for Casey House clients who do not use drugs	50 (6)
Harm reduction increases crime and other neighbourhood problems	25 (3)
Harm reduction services can cause liability issues for Casey House	25 (3)
A hospital is not a place for harm reduction	17 (2)
Harm reduction services encourage drug use	8 (1)
These services are not needed at Casey House	8 (1)
Drug treatment should be the only goal promoted at Casey House	8 (1)
Other	33 (4)

*Could select multiple responses

The interview column is omitted since no interview participants reported being less inclined to donate or undecided about the impact of new hospital-based harm reduction services on their future support (see Table 5)

When discussing the potential impact of implementing new harm reduction services at Casey House on future donor support, some interview participants worried that “other” donors might be opposed to harm reduction, due to the ongoing stigma and discrimination of PWUD in society:

*I think we should be fully on board with it. But I suspect there will be… some donors, who probably aren't… You know, just the nature of our society where there's this whole stigma around drugs, and you really shouldn't be doing it... I'm sure there's some donors who won't approve of that.* [DS115]

*If you present something to a quotation marks 'legacy donor' as harm reduction services, probably nine times out of ten, they're going to... express some support: 'That sounds… good, and proactive and that's something that I would want to be a part of.' Once you specify that that would also include safe injection sites and clean needles and supervised injections, there is a segment of the population that is going to recoil at that. Now, why is that? Well, because there will be people who believe that that is encouraging or promoting drug use.* [DS056]

Although interview participants tended to believe that this group of unsupportive “other” donors would most likely be small, nevertheless some offered to donate more to counteract the potential loss of funding. As one donor explained:

*Because of all that we've talked about, it may actually encourage me to increase my support. […] Because I am fully supportive of these types of services being offered, freely and widely. And I guess because I would be a little concerned that there may be some pushback in parts of the community that may result in certain donors deciding to discontinue or pull back on their level of support.* [DS056]

Likewise, another donor talked about going out of his way to direct his support specifically to harm reduction services if these were going to be a “hard sell” for engaging the support of “other” donors:

*I haven't requested my donations to be streamed into any particular area. That may be something I would consider, requesting that a donation I made would be put into those harm reduction services… That would be something I might consider, especially if it's going to be a hard sell…So that's how I see, maybe I could meaningfully contribute to that type of program.* [DS105]

Interview discussions also provided nuance about the appropriate roles of government funding and donor support for harm reduction services. Some claimed that harm reduction should be government funded, but until that was a reality there was a role for donors to play in ensuring these critical services could continue to be provided:

*I think the government should be funding more than they are. […] But, given that they're not, I think donors should sort of step up, and fill the gaps, where we know that the healthcare service is required, and the government doesn't seem to be doing much about it*. [DS069]

*It probably should be fully government funded, but because it's not, then, donors are forced to step up… In terms of the harm reduction… to me, it should be fully funded by the government. But obviously, if it's not, then… the onus is on the public to step up, unfortunately, right? … It's not fair to put the burden on the clients. It's not fair for clients to be left un-helped either.* [DS078]

Beyond offering financial support, participants also discussed the opportunity for Casey House to engage in education and advocacy around harm reduction, and to involve donors in this process. As one participant explained, *I think there’d be a lot of opportunity for Casey House to do education to the public about harm reduction and safe injection sites, and I think that’s extremely valuable* [DS94]. Due to ongoing stigma of substance use and a lack of knowledge, some participants thought that “other” donors and the public did not understand the need for, and value of, harm reduction services for Casey House clients:

*Yeah, I really think that it’s an issue of education, that you really have to show them* [other donors] *why this* [harm reduction] *is valuable. It should be obvious, I would think. But you know, again, we live in a society that stigmatizes illegal drug use* [DS115]

*I think the, the connection for some patients and clients, at Casey House, between their drug use and their overall health and… the quality of their lives, I'm not sure that there's a connect for many people* [public] *in that…. So, education is important, right, to see where these things are connected… drug use, of all sorts, is a part of many people's lives, including those with HIV/AIDS… I'm not sure that most people would see a direct link there, and that… will probably be a part of the Casey House approach to educating people about that.* [DS105]

However as one of these donors goes on to say, *if Casey House can reach out to their donors and explain why this is essential it will allay… some people's concerns* [DS115]. Moreover, by educating their donors, Casey House has the chance to build capacity for them to become advocates for harm reduction. As one participant describes the potential for donors sharing harm reduction information *with friends, family, business and professional colleagues*:

*With the information that Casey House provides to donors, they're also in a position to educate other people, pass on the word about what's being done… what the concerns are of people who are HIV infected, and what's being done to* [help]. […] *I mean, one of the things that Casey House always tried to do, is to sort of be on the leading edge of providing care. So, for the donors, then there's the opportunity to feel like 'Well, I can participate in that and I know about it… and I might have information that I wouldn't have otherwise, that I can share with other people...* [DS094]

### Impacts of the interview

We recognized that a few significant impacts of the interview process emerged. Participating in the study encouraged some donors to start thinking about harm reduction and exploring the programming offered at Casey House:

*Well, I hadn't really thought about it, until… your project came along. But, I know that… people who are exchanging needles are at risk for HIV and I've just read the newspaper articles about what's being done in terms of providing clean needles in places, and supervised injection sites, and that sort of thing. And it seems to be a very good direction to go, in terms of providing care for people with problems with addictions.* [DS094]

*Just to tell you the truth, when I knew I was going to do this interview, I went on the Casey House website, and there's a calendar there, from March* [2020] *obviously pre-pandemic, of all of the activities happening every day in the day health program… I was in very pleasant shock at how active that calendar was, and how much support, and how many resources are provided to people involved in the day health program. So that’s a piece of education for me, that was prompted by me knowing that I would be speaking to you.* [DS056]

The majority of survey participants felt that their patronage was appreciated/recognized “extremely well” (67%; *n* = 68) or “somewhat well” (27%; *n* = 27) (see Table 2). This research study seemed to help in this respect, as interview participants voiced how they felt valued in being consulted by Casey House on future changes to services: *[I] applaud you—not many of the other service organizations have gone to this level of sitting down there and talking to their donors at this point, about where their next steps were going to be* [DS114].

Moreover, participants emphasized the value in investing in this kind of research and of gathering data about donor opinions regarding harm reduction, given that this information may be used to help the hospital in the future. As one participant explained:

*That's why I think this kind of study is really good, and this work is really good, because it's really going to maybe show some of the effects of that. I think with knowledge is always power. And some donors may, with that knowledge, donate more or maintain their donation, in support. And, others may step back, because of certain vulnerabilities. And maybe the important part is that we provide opportunity for them to voice those reservations or those vulnerabilities and then see how we can address them at that time.* [DS125]

## Discussion

We documented widespread support of hospital-based harm reduction services among donors. The findings suggest that implementation of harm reduction programs may not adversely impact donations to Casey House. Our findings provide a novel look at donor opinions regarding support for hospital-based harm reduction services. At the time of writing no other studies, to our knowledge, have examined philanthropic donors’ acceptance/perspectives of harm reduction programs in a healthcare setting. Our research showed a deep personal connection of donors to Casey House. Study participants trusted the hospital and believed in and understood its work to improve the HIV treatment cascade and provide services for people living with HIV/AIDS, especially those who may feel marginalized in the healthcare system (e.g. LGBTQ+). This is not always the case, as other studies have found apprehension among donors, even high level/major gifts philanthropists, about the impact of their donation [52]. Often at the root of this apprehension is a difficulty in determining the credibility of an organization, a lack of transparency regarding the use of donations, and/or difficulty finding/understanding evidence about the organizations’ work/impact [52–54]. Trust in an organization and connection to its mission are key for garnering support for innovative programming, especially if this programming is a departure from its existing work. Our data shows that asking for support for harm reduction services is feasible in this context. Moreover, donors appreciated being consulted and given insight into the organization’s future endeavours.

We heard concerns about providing harm reduction services on site and how this might bring some resistance, including possible impact on funding and/or donations. Specifically, participants spoke about how “other” donors may not accept harm reduction services because they may harbour conservative/negative views of PWUD. Although it might be that participants were voicing sentiments that they heard through media or from family/friends, it would be interesting for future research to explore if these reflections are a form of externalizing or projection, where donors attribute their own doubts or unresolved emotions about substance use onto “others”. In psychology, projection involves seeing others as having traits or holding beliefs that one is suppressing or that one inaccurately considers oneself not to possess [55, 56], while externalizing involves a “tendency to project into the external world” one's beliefs, wishes, conflicts, stresses, moods, and ways of thinking [57]. Future research could examine if donors use these types of defence mechanism to deal with a lack of knowledge about harm reduction, discomfort with their own preconceived notions about substance use, or as ways to express potential reservations.

Globally, funding for harm reduction services continues to fall short of the current need, with most governments prioritizing law enforcement for dealing with substance use [6, 7, 58, 59]. Harm reduction programs are often dependent on unstable funding from a broad range of national and/or local sources which are continually impacted by political decisions [58, 60]. For example, in the USA there has been “fierce political resistance” to the implementation and scale-up of harm reduction programs which resulted in a ban on federal funding for such programs that has only been partially repealed [13, 58, 61]. Currently, harm reduction services in Canada lack sustainable political support and funding, remaining at the whim of federal, provincial, and territorial governments [62]. For instance, in Ontario, Canada’s most populous province, only needle and syringe distribution is mandated, while other harm reduction services have no steady source of government support [63]. This is problematic as changes in government can have profound implications, not only in allocation of funding, but also in indirect/direct obstruction to the implementation and operation of community-based harm reduction programs. In 2007, the newly elected conservative federal government of Canada dropped harm reduction from the Federal Drug Strategy (which has since been restored by the current liberal government) and focused on a punitive approach to substance use, bringing direct opposition to harm reduction programs across the country [64, 65]. More recently provincial governments in Ontario and Alberta pulled funding from, and pushed for closures of, SCS, and created legislation making these programs increasingly difficult to access/operate [65, 66]. The lack of consistent government commitment to funding harm reduction threatens long-term sustainability and results in tremendous variation in access to programs across jurisdictions, especially in remote and rural locations, or specific settings like hospitals and prisons.

Donor dollars are fundamental for keeping harm reduction sustainable at an organizational level. This is particularly true at a grassroots level, where most harm reduction programs start as activist/community led initiatives [67–69] and where volunteers donate their time and money to ensuring that PWUD have access to services. Donors are especially keen to avoid their donations becoming a substitute for government spending, especially when it comes to issues of health and social welfare as research shows that most people see meeting these needs as the role of the government rather than philanthropy [29]. Thus, there is a need to clearly communicate the importance of private donations for sustaining these services in the absence of consistent public funding [70]. Although many interview participants in our study saw harm reduction as a health service that should be the governments’ responsibility to fund, they also saw a role for donors to step in and fill gaps in the meantime. Indeed, some study participants spoke about finding value/meaning in being part of the harm reduction movement and ensuring life-saving services were available to hospital clients.

The ongoing criminalization and stigmatization of substance use and PWUD can make it challenging to garner support for harm reduction services, which are seen by some members of society as controversial [6, 13]. However, recent research shows that support for harm reduction is growing, with 64% of Canadian adults reporting support for a harm reduction approach to substance use, with outreach (79%), naloxone distribution (72%) and drug checking interventions (70%) receiving the highest levels of support; needle and syringe programs (60%) and SCS (55%) falling somewhere in the middle; and opioid agonist treatment (49%) and safer inhalation kits (44%) receiving the least support [71]. Thus, given that public opinion is slowly changing towards harm reduction, it may be an optimal time for more education and advocacy. Our research shows that there may be opportunity for education and capacity building among donors. For one, our findings of lower levels of support for prescription opioid treatment provide an opportunity for the hospital to educate donors about how programs like methadone, heroin-assisted treatment, and safer opioid supply programs all provide access to a safe regulated source of drugs to people, thereby reducing their risk of overdose from the toxic street drug supply. Moreover, engaging donors in honest dialogue about how harm reduction services preserve public health and safety and meaningfully impact the lives of PWUD may be a key avenue for gaining future support [13]. Indeed, research shows that providing reliable information is important for fostering altruism among donors [72]. Interview participants wanted more information regarding harm reduction and its impact on clients/community, not only for their own understanding, but also to share within their social networks. Given that some donors may have social network connections with political power or may hold such influence themselves, empowering donors by building capacity to advocate for harm reduction may be an effective way of securing additional, if not wider, support. Indeed, philanthropic donors have an opportunity to engage in ways that can influence public policy and confront inequalities faced by people who use drugs, potentially resulting in much needed social change [73].

Our study has several limitations. Although this research provides a novel look at donor support for hospital-based harm reduction programing, including nuanced and data-rich qualitative findings, our survey had a low response rate (8.2%). Thus, this sample may not be generalizable to all Casey House donors. However, response rates for online surveys tend to be lower than other survey modes [74], and the average response rate for non-profit emails ranges from 0.10% for a fundraising email to 3.6% for an advocacy email [75]. Further, those who participated in the study may have been more engaged with the hospital or had more of an interest in harm reduction. No interview participants reported strongly disagreeing/disagreeing with hospital-based harm reduction services. However, given that harm reduction is a controversial issue, those opposed to these kinds of services at the hospital may have had higher incentive to voice their dissent, than those supportive of this issue. This research was also done in a very specific setting, an HIV specialty hospital which has a long history of advocacy and fundraising for HIV/AIDS, a topic which has faced significant stigma and has been considered a “controversial” or “unpopular cause” in fundraising literature [28]. Thus, donors for this specific organization may already be sensitized to the stigma that faces clients and may be more willing to support.

## Conclusions

Our study adds to the literature on philanthropic donor attitudes towards harm reduction services, and knowledge about the potential opportunities and challenges for donor engagement on this controversial issue. Organizations which are trying to do innovative work, whether large hospitals or small grassroots community programs, often worry that donors will not support their initiatives. Our findings may be reassuring for organizations considering introducing harm reduction services. Donors were not only open to learning about harm reduction and client needs, but some were also excited about sharing this information among their networks and advocating for harm reduction more broadly. Our lower response rate suggests the need to find more ways to engage donors in research to more fully gauge how decisions about programming may or may not influence donation patterns. Harm reduction funding was in jeopardy before COVID-19 [6, 7]. With the escalating overdose crisis and the ongoing pandemic exacerbating harms for PWUD, the need for accessible services is even greater. Alternative sources of funding for harm reduction programs like donor support are crucial, especially as governments remain resistant or slow to act.

## Supplementary Information

**Additional file 1:** Donor survey instrument.

## Data Availability

The datasets generated and analysed during the current study are not publicly available due to a condition of consent, as we agreed not to share data with anyone outside of those named as part of the research team. This approach is standard for consent processes in research reviewed and approved by the University of Toronto HIV Research Ethics Board. Further it is challenging to anonymize qualitative data in the contexts as described which can be identifying in nuanced ways.

## Abbreviations

AIDS: Acquired immunodeficiency syndrome
COVID-19: Coronavirus disease 2019
HIV: Human immunodeficiency virus
LGBTQ+: Lesbian, gay, bisexual, transgender, queer
NIMBY: Not-in-my-backyard
PWUD: People who use drugs
SCS: Supervised consumption services
