# Supplementary material for: Philanthropic donor perspectives about providing harm reduction services for people living with HIV/AIDS in a hospital setting
Source: Harm Reduct J. 2022 Nov 16;19:124. doi: 10.1186/s12954-022-00711-8 (PMC9668384; doi:10.1186/s12954-022-00711-8)
Supplement: Supplementary file 1 — Additional file 1: Donor survey instrument. [file 12954_2022_711_MOESM1_ESM.pdf]

Exploring Donor Perspectives on Harm Reduction Programming at Casey House
Donor survey questions

Your giving experience with Casey House

First, we'd like to learn a bit about your relationship with Casey House and your giving experience with the organization.

1) Tell us about yourself. I am a (*check all that apply*):

☐	Mail donor
☐	Online donor
☐	Telemarketing donor
☐	Monthly donor
☐	Signature event attendee/participant
☐	Leadership donor
☐	Capital campaign donor
☐	$25,000+donor
☐	Casey House staff/physician
☐	Casey House volunteer/peer
☐	Other (please specify):
☐	Prefer not to answer

2) How long have you been a supporter/donor of Casey House?

O	First time donor
O	1-5 years
O	6-10 years
O	11-15 years
O	16-20 years
O	20+ years
O	Currently do not donate
O	Prefer not to answer

3) In the past 12 months approximately how much did you donate to Casey House?

O	Did not donate in the past 12 months
O	$1- $49 years
O	$50 - $99
O	$100-$249
O	$250 - $499
O	$500 - $999
O	$1000-$4999
O	$5000 - $9999
O	$10,000-$14,999
O	$15,000-$19,999
O	$20,000-$24,999
O	$25,000+
O	Prefer not to answer

Donor survey questions

4) What motivates you to support Casey House? (*Check all that apply*)

☐	I feel compassion for people living with HIV/AIDS and desire to make a contribution to this community
☐	I believe in Casey House and its cause
☐	Casey House has helped/is helping a family member/friend of mine
☐	I live geographically close to Casey House
☐	I work geographically close to Casey House
☐	Tax benefits
☐	I work at Casey House
☐	I volunteer/provide peer support at Casey House
☐	Other (please specify):
☐	Prefer not to answer

5) Do you feel your support is recognized and appreciated by Casey House?

O	Extremely
O	Somewhat
O	Neutral
O	Not very
O	Not at all
O	Prefer not to answer

6) Casey House provides many services to people living with HIV/AIDS. Which of the following are you aware of? *(Check all that apply)*

☐	**Respite inpatient care** (short-term beds available for clients accessing specialized healthcare, frequently when recovering from a surgery or other health complications related to HIV/AIDS, and care-giver support)
☐	**Rehabilitative inpatient care** (consists of pain management, medication adherence, symptom control, post-hospital discharge support)
☐	**Palliative/end of life care**
☐	**Day health program** (includes clinical services and community programs such as: yoga, nursing clinic, meditative art, book club, diabetes workshop, daily lunch, cooking group, foot care clinic, afternoon movie, harm reduction kit making, seeking safety group, music therapy, haircuts, etc.)
☐	**Harm reduction kit distribution** for clients and the surrounding community
☐	**Child care fund** (provides access to child care funding for any parents living with HIV with children under the age of 12. The funds are specifically geared to helping parents at times when they are ill/hospitalized, need respite care, or are attending healthcare appointments, workshops and support groups. The funds are available to Casey House clients as well as any parents living with HIV in the greater Toronto area)
☐	**Managed alcohol services** (a harm reduction service part of the inpatient program aimed at preventing some of the harms of severe alcohol dependence. Clients are provided with tailored amounts of beverage alcohol as a means to safely stabilize or reduce alcohol consumption, and reduce harms by replacing non-beverage alcohol (e.g., mouthwash, hand-sanitizer, hairspray) with less harmful beverage alcohol)
☐	**Volunteer and peer programs** (volunteers and peers with lived experience with HIV/AIDS offer their time, skills, and talent to support Casey House clients in a variety of ways in one-on-one or group activities)
☐	Other (please specify):
☐	Prefer not to answer

Donor survey questions

Harm Reduction Knowledge

Next, we wish to learn about your knowledge of, and opinions about harm reduction programming, including services that are being currently offered and those that are being considered at Casey House.

7) How much do you know about harm reduction? (*Check the closest answer*)

O	**Very little or no knowledge** *(I haven't heard the term before or I know the term but not a lot about what's included)*
O	**Some knowledge** (I know something about services or programming offered)
O	**Average knowledge** (*I know something about programming/services offered and about evidence of the effectiveness of a harm reduction approach for people who use drugs)*
O	**Fairly knowledgeable** (*I know details about programming/services offered and evidence about the effectiveness of a harm reduction approach, have followed the news about setting up new harm reduction services in Toronto/across Canada)*
O	**Very knowledgeable** *(I know details about programming offered and evidence supporting harm reduction, have followed the news about setting up new harm reduction services, have either done some extra reading or know people who work at or have used a harm reduction program/service)*
O	Prefer not to answer

8) Harm reduction services are offered in a variety of locations across Canada. Which general types of harm reduction services have you heard of before? (*Check all that apply*)

☐	Harm reduction kit distribution
☐	Needle and syringe programs
☐	Supervised injection services (SIS)
☐	Supervised consumption services (SCS)
☐	Opioid agonist therapy (OAT)/opioid substitution/maintenance treatment (e.g., methadone)
☐	Injectable opioid agonist therapy (iOAT)
☐	Safer drug use education
☐	Naloxone kit distribution
☐	Other (please specify):
☐	Prefer not to answer

Definition of harm reduction

When we speak of harm reduction, we refer to policies, programmes and practices that aim to reduce the harms associated with the use of drugs. Harm reduction is built on a belief in, and respect for, the rights of people who use drugs. A harm reduction approach works with people without discrimination, judgement, coercion, or requiring that they stop using drugs as a precondition of support. Harm reduction incorporates a spectrum of strategies from safer use, to managed use, to abstinence (definition adapted from International Harm Reduction Association and Harm Reduction Coalition).

Casey House is working on implementing more evidence-based harm reduction programming, given that these services have the potential to improve the health and well-being of clients, reduce overdose and other drug-related harms, increase antiretroviral therapy adherence, and connect vulnerable clients to care.

<u>Opinions about harm reduction programs</u>

Whatever your current level of knowledge about harm reduction, in the next questions, we will ask you about three specific types of harm reduction programs and provide a definition of each.

Donor survey questions

Harm Reduction Kit Distribution

Many community health centers, public health units and other organizations that serve people who use drugs distribute harm reduction kits. These kits are available for free and provide safe, sterile supplies (e.g., needles, syringes, cotton, cooker, water, glass stem (for crack/meth pipes), condoms, etc.). The goal of kit distribution is to reduce infectious disease transmission, health complications, and other injuries associated with drug use. Casey House has been distributing harm reduction kits to its clients and the surrounding community since 2014.

9a) Do you agree or disagree with making **harm reduction kits** available as part of **overall health care in Ontario** for people who use drugs?

O	Strongly agree
O	Agree
O	Undecided
O	Disagree
O	Strongly disagree
O	Prefer not to answer

9b) Do you agree or disagree with making **harm reduction kits** available **at Casey House** for clients and the surrounding community?

O	Strongly agree
O	Agree
O	Undecided
O	Disagree
O	Strongly disagree
O	Prefer not to answer

Supervised Consumption Services (SCS)

Supervised consumption services are health services that provide a hygienic space for people to come and use their pre-obtained drugs under the supervision of trained staff. In addition to supervised use, individuals are provided with sterile equipment (needles, syringes, cotton, cooker, water, etc.), education on safer use, overdose prevention and intervention, as well as referrals to various health and social services (e.g., counselling, treatment, housing, income support). The goals of SCS are to decrease the transmission of diseases, reduce overdose deaths, minimize public drug use, and improve access to health and social services. Casey House has support from the board to offer supervised consumption services to clients only and is currently working to implement supervised injection and potentially other routes of consumption in the future.

10 a) Do you agree or disagree with making **supervised consumption services** as part of **overall health care in Ontario** for people who use drugs?

O	Strongly agree
O	Agree
O	Undecided
O	Disagree
O	Strongly disagree
O	Prefer not to answer

10b) Do you agree or disagree with making **supervised consumption services** available **at Casey House for clients** who use drugs?

O	Strongly agree
O	Agree
O	Undecided
O	Disagree
O	Strongly disagree
O	Prefer not to answer

<u>Prescription Opioid Treatment</u>

Opioids are psychoactive drugs typically used to relieve pain, and include prescribed pain medication (e.g. codeine, morphine, Percocet) and illegal drugs (e.g., heroin). Prescription opioid treatment involves physicians or nurse practitioners prescribing doses of opioids in injectable or tablet form (which is self-administered by the client under supervision). The goal of prescription opioid treatment is to treat opioid use disorder by providing clients with access to opioids that are of a known dosage and quality to prevent withdrawal and reduce their exposure to contaminated drugs bought through illegal markets. Such programs (e.g., injectable opioid agonist therapy (iOAT), safe supply) are operating in Western Canada in several locations (e.g., Royal Alexandra Hospital (Edmonton), Dr. Peter Centre (Vancouver)). Casey House does not currently offer this service but is carefully considering this evidence-based approach for its clients.

11a) Do you agree or disagree with making **prescription opioid treatment** available as part of **overall health care in Ontario** for people who inject drugs?

O	Strongly agree
O	Agree
O	Undecided
O	Disagree
O	Strongly disagree
O	Prefer not to answer

11b) Do you agree or disagree with making **prescription opioid treatment** available **at Casey House for clients** who inject drugs?

O	Strongly agree
O	Agree
O	Undecided
O	Disagree
O	Strongly disagree
O	Prefer not to answer

Impact of Harm Reduction Programming on Your Support

12) How, if at all, will Casey House implementing more harm reduction services for its clients (e.g., SCS, iOAT) impact your future donations to Casey House?

O	I will be less inclined to donate to Casey House
O	I will be more inclined to donate to Casey House
O	It will not impact how I donate to Casey House
O	Haven’t made up my mind
O	Prefer not to answer

Donor survey questions

[Only ask if for Q12 participant answered: less inclined to donate OR haven't made up my mind]

13) Please *check all* the reasons why you might be less inclined to donate:

☐	Harm reduction services encourage drug use
☐	These services are not needed at Casey House
☐	A hospital is not a place for harm reduction services
☐	Harm reduction services increase crime and other neighbourhood problems
☐	Harm reduction services can cause liability issues for Casey House
☐	Harm reduction services will take away from the focus on HIV/AIDS at Casey House
☐	Harm reduction services are out of line with the care and services that I believe Casey House should provide
☐	Drug treatment should be the only goal promoted by Casey House for clients who use drugs
☐	Abstinence should be the only goal promoted by Casey House for clients who use drugs
☐	Providing harm reduction services would disrupt care for Casey House clients who do not use drugs
☐	Other (please specify):
☐	Prefer not to answer

[Only ask if for Q12 the participant answered: more inclined to donate OR haven't made up my mind]

14) Please *check all* the reasons why you might be more inclined to donate:

☐	Harm reduction services encourage safer drug use
☐	The current opioid overdose crisis warrants the introduction of harm reduction services at Casey House
☐	A hospital/day health program are ideal locations for harm reduction services
☐	Harm reduction services reduce crime and other neighbourhood problems
☐	Harm reduction services reduce overdose deaths and infectious disease transmission (e.g., hepatitis C, hepatitis B, etc.)
☐	Harm reduction services can increase contact of clients who use drugs with health and social workers
☐	Harm reduction services can help to retain Casey House clients in HIV care/treatment
☐	Harm reduction services are in line with the care and services that I believe Casey House should provide
☐	Other (please specify):
☐	Prefer not to answer

15) How would you prefer to learn about new harm reduction initiatives at Casey House? (*Check all that apply*)

☐	Through an educational session
☐	At a town hall meeting/public forum
☐	By phone/private meeting
☐	I don't want to hear about new initiatives
☐	Through an educational session
☐	Other (please specify):
☐	Prefer not to answer

<u>Sociodemographic Questions:</u> Finally, we wish to ask you a few questions about yourself, for example your age, cultural background, marital status in order to get a broader picture of Casey House donors.

16) Do you identify as:

O	Male
O	Female
O	Other, please specify:
O	Prefer not to answer

17) What is your age?

O	Under 18 years old
O	18 - 34 years old
O	35 - 54 years old
O	55 - 74 years old
O	75 years or older
O	Prefer not to answer

Donor survey questions

18) Where do you currently reside?

O	In the greater Toronto area (GTA)
O	Within Canada (but outside GTA)
O	Outside of Canada
O	Prefer not to answer

19) How would you identify your racial and cultural background? (*Please check as many as apply*)

O	**Indigenous** (i.e., origins in First Nation, Metis, Inuit, etc.)
O	**Asian** (South Asian, East Asian, Central Asian, South East Asian, Western Asian) **(** i.e., origins in Cambodia, China, India, Bangladesh, Japan, Korea, Malaysia, Pakistan, the Philippine Islands, Thailand, and Vietnam)
O	**African, Black or Caribbean** (i.e., origins in any of the black racial groups of Africa)
O	**Middle Eastern** (i.e., origins in the Middle East, North Africa) White/Caucasian (i.e., origins in any of the original peoples of Europe)
O	Other, please specify:
O	Prefer not to answer

20) We would also like to learn more about your connection to Casey House and substance use. *Click all of the following that are true for you*:

☐	I donate only to Casey House
☐	I donate to other charities besides Casey House
☐	I volunteer in my community
☐	I have drank alcohol at least once (ever)
☐	I have used cannabis at least once (ever)
☐	I have used other drugs at least once (ever)
☐	I have worked with people who use drugs in a professional capacity
☐	I have worked with people who use drugs in a volunteer capacity
☐	I currently live in close proximity to Casey House
☐	I currently work in close proximity to Casey House or I currently work at Casey House
☐	I sometimes attend charity events at Casey House
☐	I often attend charity events at Casey House
☐	Prefer not to answer

21) What is the highest level of schooling you have completed?

O	High school or less
O	College or university
O	Graduate or professional school
O	Other (please specify):
O	Prefer not to answer

22) Currently, what is your marital status? (*Please click one answer*)

O	Married/living with a partner
O	Widowed
O	Divorced/separated
O	Single/never married
O	Prefer not to answer
